# The Effects of Birth Weight and Maternal Care on Survival of Juvenile Steller Sea Lions (*Eumetopias jubatus*)

**DOI:** 10.1371/journal.pone.0096328

**Published:** 2014-05-07

**Authors:** John M. Maniscalco

**Affiliations:** 1 Department of Science, Alaska SeaLife Center, Seward, Alaska, United States of America; Université de Sherbrooke, Canada

## Abstract

Steller sea lions were listed as endangered following a collapse of the western distinct population beginning in the late 1970s. Low juvenile survival has been implicated as a factor in the decline. I conducted a multistate mark-recapture analysis to estimate juvenile survival in an area of the western population where sea lions are showing signs of recovery. Survival for males and females was 80% between 3 weeks and 1 year of age. Approximately 20% of juveniles continued to be nursed by their mothers between ages 1 and 2 and 10% between ages 2 and 3. Survival for juveniles that suckled beyond 1 year was 88.2% and 89.9% to ages 2 and 3, respectively. In contrast, survival for individuals weaned by age 1 was 40.6% for males and 64.2% for females between ages 1 and 2. Birth mass positively influenced survival for juveniles weaned at age 1 but had little effect on individuals continuing to suckle. Cumulative survival to age 4 was double that estimated during the population decline in this region. Evidence suggests that western Steller sea lions utilize a somewhat different maternal strategy than those in the eastern distinct population. Western adult females generally invest more in their pups during the first year but wean offspring by age 1 more often. This results in better survival to age 1, but greater mortality between ages 1 and 3 compared to the eastern population. Different maternal strategies may reflect density dependent pressures of populations at opposite levels of abundance.

## Introduction

Juvenile survival is an important life history variable affecting population growth and can be greatly influenced by environmental variation in large iteroparous mammals [1], [2]. Environmental factors affect maternal body condition, health, and pregnancy status which, in turn, can affect reproductive rates and juvenile survival [1], [3], [4]. The quality and extent of maternal care among mammals can be measured by attentiveness to offspring needs in the form of nurturing or nursing, and subsequent survival of those offspring. The stage at which offspring are weaned and become independent has limited flexibility among most mammals and may depend on a complex interplay of parent and offspring needs with respect to available resources among other things [3]–[5]. Variation in the duration of maternal care and nursing reaches an extreme among otariid pinnipeds (fur seals and sea lions) where it can vary between 8 months and 4 years for certain species [6], [7]. Yet, there have been no direct measures of the effect that continued maternal care has on survival rates among juvenile pinnipeds.

Steller sea lions (*Eumetopias jubatus*) are the largest of the otariids and likely have the largest variation in the duration of lactational dependence [6], [8]. Females of this species become reproductively mature at 3 to 7 years of age and give birth to one pup per year but not necessarily every year [6]. Twinning is extremely rare and adult females may occasionally nurse offspring of different ages simultaneously [9].

Since the 1970s, Steller sea lions in the western distinct population segment (WDPS; [10], [11]) of the North Pacific Ocean declined by over 80% [12] and are currently listed as endangered under the Endangered Species Act of the United States. Most of the decline occurred during a catastrophic collapse spanning about 15 years between the late 1970s and early 1990s. Much research during the past two decades has been dedicated to understanding potential causal factors such as nutritional limitation due to interaction with economically important fisheries [13], [14] or climate change [15], and predation by killer whales (*Orcinus orca*; [16]). Early survival estimates for WDPS sea lions were based on age composition counts and life history tables [17], [18] and indicated that juvenile survival was reduced during the height of the population decline in the Gulf of Alaska during the 1980s compared to the 1970s. A subsequent estimate of juvenile survivorship during the period from 1987 to 1991, based on mark-recapture analysis of individuals from approximately 3 weeks of age, suggested good survival to age 1 (80%) but much lower survival for ages 1 – 2 and 2 – 3 (61% per age group; [19]). Both studies implicated low juvenile survival as a contributor to the population decline.

Steller sea lion populations and pup production have generally increased since 2001 between the eastern Aleutian Islands and Gulf of Alaska regions of the WDPS with the most strongly positive trends observed in the Gulf of Alaska [20]. This may be due in part to high natality rates of adult females in this region [21]. Improved juvenile survival may also be aiding the observed recovery. A recent estimate based on actual detection of mortalities in a small sample of juveniles between 2005 and 2011 suggested that survivorship had recovered somewhat since the 1980s to 64% for animals 1 – 2 years and 83% from 2 to 3 years [22]. Similarly, mark-recapture estimates of annual Steller sea lion survival in the eastern distinct population segment (EDPS) in southeastern Alaska range from 65% to 97% for males and females aged 1 to 4 years [23]. The EDPS has also been increasing over at least the past few decades [24], [25]. It is naturally important to understand what factors might be affecting these changes in juvenile survival.

The purpose of this study was to provide an updated estimate of pup and juvenile survival from age 3 weeks to 4 years based on mark-recapture data from the WDPS that can be compared to similar work during the WDPS decline [19] and current estimates in the EDPS [23]. Results presented in the current work cannot be clearly compared to the earliest estimates of survival based on juvenile proportions and life history models [18] because of different assumptions made between that study and this one. I also estimated the effect that birth mass and multiple years of maternal nursing had on juvenile survival along with proportions weaned at ages 2 and 3 using a multistate mark-recapture approach [26], [27]. Birth mass has often been found to have an effect on future survival in pinnipeds and terrestrial mammals [1], [23], [28], [29]. However, among mammals that exhibit large variations in maternal dependence, how long a mother nurses her offspring may have an even greater effect on future survival [7], [30], [31]. These and other covariates were tested among Steller sea lions in this study to gain a better understanding of how female life history choices can affect juvenile survival in this endangered species.

## Materials and Methods

### Ethics Statement

This research was conducted in accordance with Alaska SeaLife Center Institutional Animal Care and Use Committee Protocol No. R10-03-01 and National Marine Fisheries Service Permit No. 14324 for research on endangered Steller sea lions. The Chiswell Island group is part of the U. S. Fish and Wildlife Service National Maritime National Wildlife Refuge. Research was conducted on Refuge lands under right-of-way Permit No. M-344-AM and Special Use Permit No. 74500-10-001 and earlier versions.

### Study Site and Field Methods

**Figure 1:**
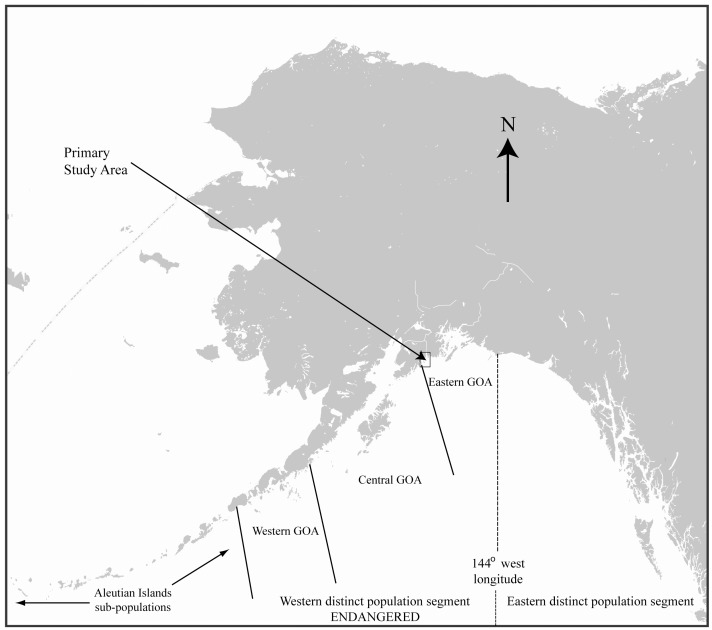
Map of Alaska showing the delineation between the western (endangered) and eastern distinct population segments of Steller sea lions and the primary area of study for this research in the eastern Gulf of Alaska.

This study was centered on Steller sea lions from the Chiswell Island rookery in the eastern Gulf of Alaska, part of the endangered WDPS (Figure 1). Sea lions at this rookery and the surrounding area have been well-studied since 1999, primarily through the use of a remote video system [21], [32]. Pups were captured at the rookery on one day in each of the years 2005, 2007, 2008, and 2010 near the end of the pupping season (June 30 – July 3). Body mass was determined by weighing pups to the nearest 0.1 kg in a tared hoop net with a hanging electronic scale (FWC series 7, FlexWeigh, Santa Rosa, CA). While anesthetized in sternal recumbence on a flat board, pups were sexed, measured and permanently marked by hot-iron branding as described by Merrick et al. [33].

Age at capture in previous studies such as this has been assumed to be about 3 weeks based on time from peak birthing periods [19], [23], [34]. In this study, most adult females were individually recognizable by natural markings, brands, or tags, and monitored for timing of birth and attendance patterns [32]. Therefore, it was possible to determine the exact age of marked pups in almost all cases when they reunited with their mothers whose time of parturition was known to within±4 hrs. This assumes that mothers reunited with their own pups and not others, and was considered reasonable given that otariids form strong mother-pup bonds from a very young age [35]–[37].

Resighting efforts were conducted during systematic scan sampling as described by Altmann [38] using the remote video system based on Chiswell Island and neighboring haulouts in the surrounding area [21], [32]. These local efforts were supplemented with observations from small boats and tour vessels. Additional resightings throughout Alaska came from dedicated annual efforts by the National Marine Fisheries Service and Alaska Department of Fish & Game. Only sightings that could be verified with a photograph were used in this analysis. Behavior of each of the resighted animals was recorded with special attention to nursing activity of the mother. The annual observation window extended from 20 June through 31 October to utilize data from a broad range of sources and dedicated efforts. Observation effort also was consistent between years with number of days of effort varying <5% from all sources across years.

It was not always possible to determine if a juvenile was still suckling and with its mother beyond 1 year of age, especially when only one or few observations of the animal were recorded. Kendall et al. [39] provide a robust design method for dealing with state uncertainty such as this but that method requires a large increase in parameters being estimated. The increased parameterization combined with the relatively short duration (<7 years) of this study plus the inclusion of an individual covariate (birth mass) resulted in poor performance of many models using that approach. Therefore, a standard multistate approach was used, but with ancillary information on the location and status of the mothers, state uncertainty was greatly reduced. For example, if a juvenile was observed without its mother in any location, we would cross-check our database for the status of the mother at that time. If the mother was attending to a newborn pup on the rookery without the elder sibling, then it would be confirmation that the previously marked juvenile had been weaned. This left only 2 juveniles of unknown status and with their removal from the dataset, allowed the use of a standard multistate modeling approach rather than robust design multistate modeling.

### Data Analysis

Data were analyzed in Program MARK under a multistate design [40], [41] using the logit link function to estimate survival (S), sighting probabilities (p), and state transitions probabilities (ψ) for juveniles up to age 4. Two different states were designated as suckling (s) and independent or weaned (w). Transitions between states (ψ^ss^ and ψ^sw^) were assumed to be Markovian such that state observed at time *i* was dependent only on the state observed at time *i*-1. Transition from independence back to suckling (ψ^ws^) is rarely observed in the wild for this species (ASLC unpublished data), so was constrained to 0. Sex was included as a grouping variable and birth mass (range: 13.2 – 32.4 kg) as an individual covariate for each pup. Birth mass was estimated from linear regressions based on mass at capture versus age for each sex and cohort. The regression residuals for each pup were added to the y-intercepts to obtain the mass estimates. Estimates of birth mass by this method are considered to be of “high quality” [42].

Multinomial models were compared with an information-theoretic approach to provide a relative strength of evidence for alternative models [43], [44]. This technique uses Akaike’s Information Criteria (AIC; [45]) with an additional correction for small sample bias (AICc; [46]) to determine the best fitting model(s). The fully parameterized time-dependent model was first tested for goodness of fit (GOF) using program U-Care [47].

Other than the state transition constraint mentioned above, 2 additional constraints were placed on all fitted models, with the exception of the fully parameterized time-dependent model. First, survival was constrained to be equal for juveniles transitioning to independence and for those continuing to suckle between ages 0 and 1 because weaning typically occurs between April and mid-June in this species [8] which is outside our late-June to October observation period. In this manner, state transition was assumed to have occurred late in the non-observation period and survival was dependent only on previous state. Second, probability of sighting a suckling juvenile (p^s^) was constrained to 1 for all ages and both sexes because this value was found to be very close to 1 in preliminary analysis and had confidence intervals exceeding 1, which can cause models of this type to perform poorly [27]. Further constraints to the models were placed with regard to biological relevance in the search for the most parsimonious model(s) that provide the most information with the fewest parameters. For example, survival of suckling juveniles was constrained to be equal between the sexes for some models tested. If those models express much smaller AICc values (more parsimonious) than other models in which survival was allowed to vary between sexes, then it can be said that survival is not different between males and females that are suckling. In this manner, a variety of constraints were placed on survival, sighting probabilities, and state transitions to be tested for their effect on model fit.

Parameter estimates were obtained from averaging all models that fit the data using the modern principals of multimodel inference [44], [48]. Estimates of survival are of apparent survival because actual deaths could not be differentiated from permanent emigration. Combined survival estimates for all individuals at each age were determined from proportions estimated in each state and sex category with error calculated using the Delta Method of R. Dorfman [49] with variance-covariance matrices provided from Program MARK. Calculation of the proportion of juveniles that were suckling at different ages was performed using equation 2 in Nichols et al. [50] with corresponding estimates of error.

## Results

**Figure 2:**
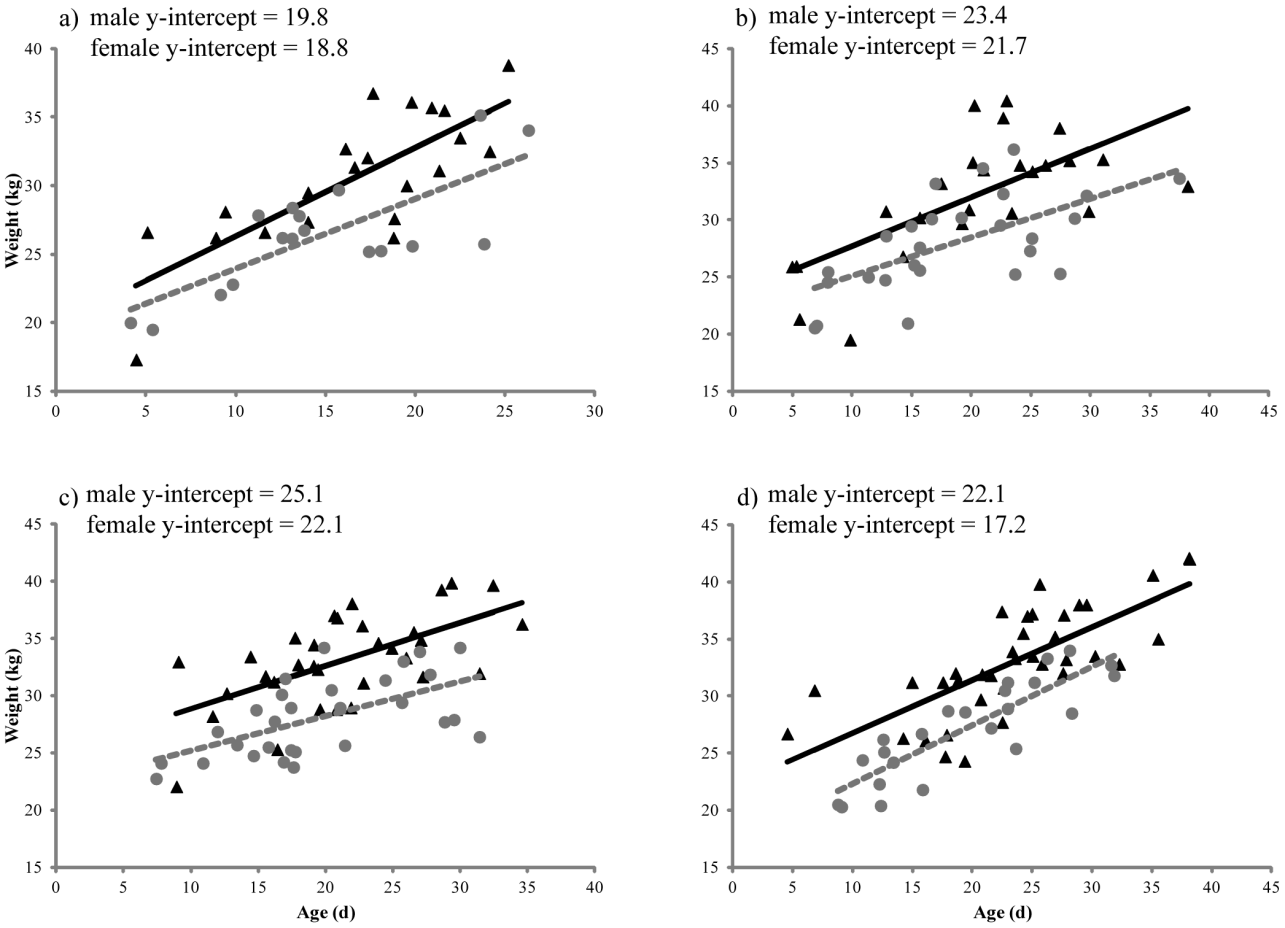
Regressions of mass on age for males (▴) and females (•) from a) 2005, b) 2007, c) 2008, and d) 2010. Residuals for each individual were subtracted from the y-intercept by sex and year to obtain birth mass estimates. All regressions were highly significant (P <0.001).

A total of 199 pups over the 4 cohorts were captured, weighed, and observed at least once with their mothers whose time of parturition was known. Pups in this study ranged from 5 – 38 days old at time of capture and were close to 3 weeks on average (19.8±0.51 d). All regressions for mass-at-age of the neonate pups were highly significant (P<0.001) for each sex and cohort, providing reasonable estimations of mass at birth (Figure 2). Mass at birth ranged from 13.2 to 28.2 kg for females (n = 89) and 14.0 to 31.4 kg for males (n = 108). Two pups could not be positively identified with their mother and were assigned the mean estimated birth mass based on the regressions for their sex and birth year. This method provides accurate representation of relatively small proportions (1% in this case) of missing data [51]. Resightings of juveniles were concentrated within a few hundred km of their birth location at Chiswell Island. However, a few individuals ranged as far west as the Alaskan Peninsula and as far east as Glacier Bay in the EDPS, ca. 800 km in either direction. Movement of these and many other marked Steller sea lions in Alaska were examined in another study [52].

Based on GOF tests of the full model, there was an insignificant degree of overdispersion with regard to the effect of past encounter history and with regard to capture probability for individuals known to be alive (ĉ = 1.27, *P* = 0.298). Therefore, no overdispersion estimate was applied to AICc values.

In addition to the fully time dependent model, 35 additional models were fitted with various logical constraints on all parameters examined (Table 1). Effects of time and cohort on survival, sighting probability, and state transition were not well supported by the data. Models with sighting probabilities for independent juveniles (p^w^) varying between ages 1 – 4 and with sex differences were better supported than those with equality between the sexes. As noted in the methods, sighting probabilities for suckling juveniles (p^s^) were close to 1, and therefore constrained to 1, and were not different between the sexes. Sighting probabilities ranged from about 32% to 100% for independent male and female juveniles and generally increased with age (Table 2).

**Table 1:** Parameter structure for multistate models fit to the data for this study.

Model	AICc	ΔAICc	AICc weight	Model Likelihood	No. Param	Deviance
S^s^(age1,2)S^w^(mass+sex*age2,3) p(sex*age1–4) ψ(age1–3)	970.7572	0.0000	0.2225	1.0000	20	928.6520
S^s^(age1,2)S^w^(mass+sex*age2,3) p(sex*age1–4) ψ(age1–5)	971.3764	0.6192	0.1633	0.7337	22	924.8273
S^s^(age1,2)S^w^(sex*age2,3) p(sex*age1–4) ψ(age1–3)	971.8922	1.1350	0.1262	0.5669	19	931.9923
S^s^(mass+age1,2)S^w^(mass+sex*age2,3) p(sex*age1–4) ψ(age1–5)	972.3526	1.5954	0.1002	0.4504	23	923.5647
S^s^(age1,2)S^w^(sex.age2,3) p(sex*age1–4) ψ(age1–5)	972.4809	1.7237	0.0940	0.4224	21	928.1593
S^s^(age1–3)S^w^(mass+sex*age2,3) p(sex*age1–4) ψ(age1–5)	973.4344	2.6772	0.0583	0.2622	23	924.6465
S^s^(age1–3)S^w^(mass+sex*age2–4) p(sex*age1–4) ψ(age1–5)	974.3340	3.5768	0.0372	0.1672	25	921.0345
S^s^(age1–3)S^w^(sex*age2,3) p(sex*age1–4) ψ(age1–5)	974.5155	3.7583	0.0340	0.1527	22	927.9664
S^s^(age1–3)S^w^(sex*age2–4) p(sex*age1–4) ψ(age1–3)	974.8220	4.0648	0.0292	0.1310	22	928.2729
S^s^(age1–3)S^w^(mass*sex*age2,3) p(sex*age1–4) ψ(age1–5)	975.4173	4.6601	0.0217	0.0973	24	924.3793
S^s^(age1–3)S^w^(sex*age2–4) p(sex*age1–4) ψ(age1–5)	975.4387	4.6815	0.0214	0.0963	24	924.4007
S^s^(age1–3)S^w^(mass+sex*age2–4) p(sex*age1–4) ψ(mass+age1–3)	975.4490	4.6918	0.0213	0.0958	24	924.4110
S^s^(mass+age1–3) S^w^(mass+sex*age2,3) p(sex*age1–4) ψ(age1–5)	975.6248	4.8676	0.0195	0.0877	25	922.3254
S^s^(age1–3)S^w^(mass*sex*age2–4) p(sex*age1–4) ψ(age1–5)	976.1670	5.4098	0.0149	0.0669	26	920.5944
S^s^(age1–3)S^w^(sex*age2–4) p(sex*age1–4) ψ(sex*age1–3)	976.3734	5.6162	0.0134	0.0603	25	923.0740
S^s^(age1–3)S^w^(mass*sex*age2–4) p(sex*age1–4) ψ(mass+age1–3)	977.2996	6.5424	0.0085	0.0380	25	924.0001
S^s^(mass+age1–3)S^w^(mass*sex*age2–4) p(sex*age1–4) ψ(mass+age1–3)	978.4781	7.7209	0.0047	0.0211	26	922.9056
S^s^S^w^(age1–3) p(age1–4) ψ(age1–5)	979.0841	8.3269	0.0035	0.0155	14	950.0471
S^s^(age1–3)S^w^(sex*age2–4) p(age1–4) ψ(age1–3)	980.0261	9.2689	0.0022	0.0097	18	942.3203
S^s^S^w^(age1–3) p(sex*age1–4) ψ(age1–5)	980.4348	9.6776	0.0018	0.0079	18	942.7291
S^s^(age1,2)S^w^(mass+sex+age2,3) p(sex*age1–4) ψ(age1–3)	981.0591	10.3019	0.0013	0.0058	18	943.3534
S^s^S^w^(mass+age1–3) p(age1–4) ψ(age1–5)	981.2237	10.4665	0.0012	0.0053	15	950.0355
S^s^(sex*age1–3)S^w^(sex*age2–4) p(sex*age1–4) ψ(sex*age1–3)	981.3064	10.5492	0.0011	0.0051	28	921.1530
S^s^S^w^(sex*age1–3) p(age1–4)ψ(sex*age1–5)	987.4274	16.6702	0.0001	0.0002	22	940.8783
S^s^S^w^(age1–3) p(sex*age1–4) ψ(sex*age1–5)	988.0991	17.3419	0.0000	0.0002	23	939.3113
S^s^S^w^(sex.age1–3) p(sex*age1–4) ψ(sex*age1–5)	988.3035	17.5463	0.0000	0.0001	26	932.7309
S^s^S^w^(mass+sex*age1–3) p(age1–4) ψ(sex*age1–5)	989.2361	18.4789	0.0000	0.0001	23	940.4482
S^s^S^w^(sex*age1–4) p(sex*age1–4) ψ(sex*age1–5)	990.3418	19.5846	0.0000	0.0000	28	930.1883
S^s^(sex*age1–4)S^w^(sex*age2–4) p(sex*age1–4) ψ(sex*age1–5)	992.4237	21.6665	0.0000	0.0000	34	918.2419
S^s^(sex*age1–3)S^w^(sex*age2,3) p(sex*coh+age1–3) ψ(sex*age1–3)	1004.198	33.4405	0.0000	0.0000	40	915.5434
S^s^(sex*age1–4)S^w^(coh.sex*age1–4) p(sex*age1–4) ψ(sex*age1–5)	1009.260	38.5029	0.0000	0.0000	50	895.4389
S^s^(coh.sex*age1–3)S^w^(coh.sex*age2,3) p(sex*age1–3) ψ(sex*age1–3)	1047.411	76.6533	0.0000	0.0000	66	890.3567
S^s^(coh.sex*age1–3)S^w^(coh+sex*age2,3) p(coh.sex*age1–3) ψ(coh.sex*age1–3)	1065.931	95.1737	0.0000	0.0000	81	864.6291
S^s^(coh.sex*age1–4) S^w^(coh.sex*age2–4) p(coh.sex*age1–4) ψ(coh.sex*1–4)	1121.474	150.717	0.0000	0.0000	106	837.0011
Full model+basic constraints: age1(S^s^ = S^w^) p^s^ = 1 ψ^ws^ = 0	1242.371	271.614	0.0000	0.0000	139	825.3707
Full model	1463.161	492.403	0.0000	0.0000	181	824.3375

Ages represent separation of years with older ages than those listed being grouped as similar. S^s^ and S^w^ represent structure for survival of those that were suckling and those that were weaned in year *i* – 1, respectively. Sighting probability structure for individuals that were weaned (p^w^) is represented by p and those that were suckling (p^s^) constrained to 1. State transitions from suckling to independence (ψ^sw^) are represented by ψ, while the reverse (ψ^ws^) was constrained to 0.

**Table 2:** Model-averaged sighting probabilities and confidence intervals for weaned males and female aged 1 – 4.

Sex and Age	Sighting Prob.	Lower Conf. Lim.	Upper Conf. Lim.
Males at age 1	0.317	0.199	0.466
Males at age 2	0.608	0.420	0.768
Males at age 3	0.781	0.573	0.905
Males at age 4	0.999	0.966	1.000
Females at age 1	0.549	0.374	0.712
Females at age 2	0.667	0.499	0.801
Females at age 3	0.821	0.641	0.922
Females at age 4	0.747	0.583	0.862

Transition probabilities from suckling to weaning (ψ^ss^ and ψ^sw^) that were constrained to be equal between the sexes had better strength of evidence than those varying between the sexes indicating no difference in age at weaning for males and females. Birth mass was not favored as a contributor to age at weaning, first appearing in the 12^th^ ranked model with a ΔAICc of 4.692 (Table 1).

As anticipated, survival probabilities (S) were best represented by differences between juveniles that were suckling in year *i* – 1 and those that were weaned at *i* – 1. The best fitting model with survival set to be equal between those 2 groups ranked 18^th^ with a ΔAICc of 8.327 and a likelihood of <0.02 (Table 1). All of the best fitting models expressed some effect of sex on survival for independent juveniles (S^w^) but not for those continuing to suckle (S^s^), indicating that males and females that continued to suckle beyond 1 year of age benefitted equally. Mass was also included in most of the best fitting models as an important contributor to survival for weaned juveniles but generally not favored for an effect on survival for juveniles still suckling (Table 1).

**Figure 3:**
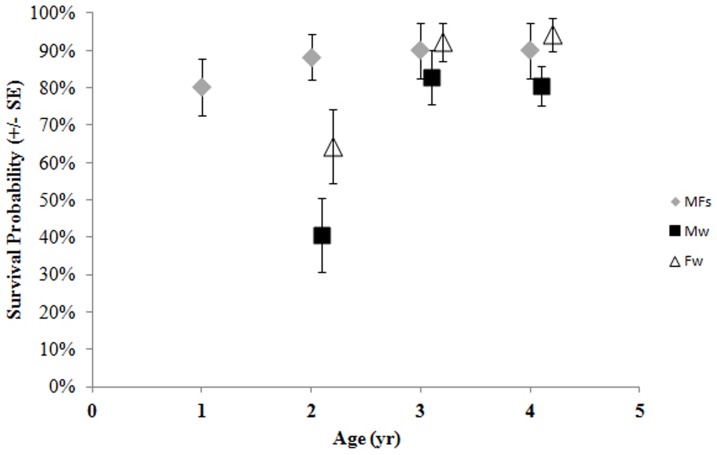
Model-averaged survival estimates (± SE) of Steller sea lions to ages 1 – 4 for suckling males and females (MFs), weaned males (Mw) and weaned females (Fw).

**Figure 4:**
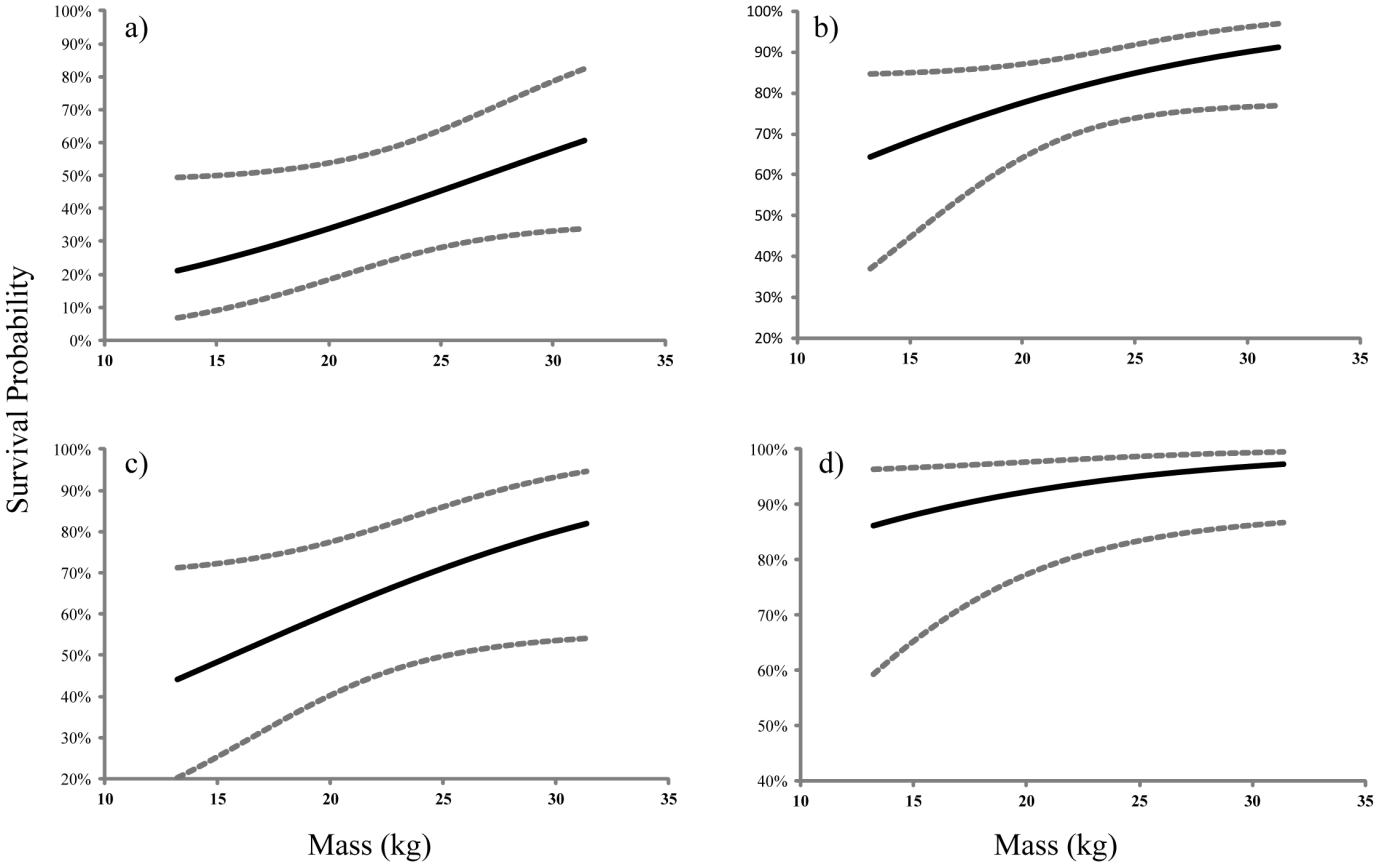
The effect of birth mass on survival for independent juveniles: a) males to age 2, b) males to age 3, c) females to age 2, and d) females to age 3. Note different y-axis scales.

**Figure 5:**
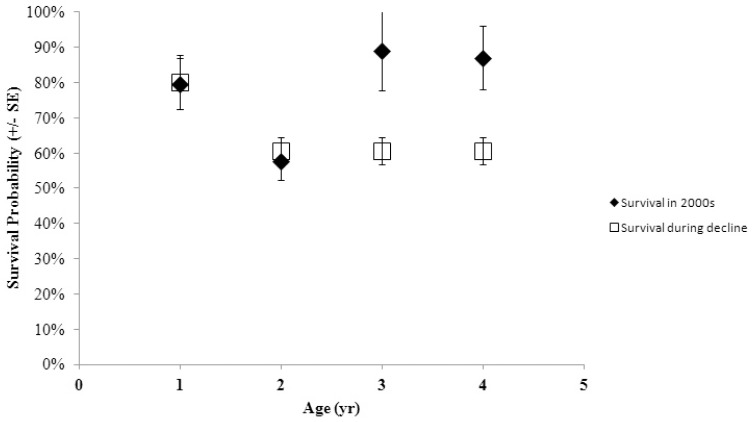
Survival probabilities for male and female juveniles at age during the period of the decline (open squares; Pendleton et al. 2006) and during the 2000s (closed diamonds; this study).

Survival to age 1 was estimated at 80.1% for all juveniles but dropped to a low of 40.5% for weaned (S^w^) males between ages 1 and 2 (Figure 3). Survival estimates also generally increased with age, especially for independent females and for males and females that continued to suckle. Estimated birth mass was positively correlated with survival for independent males and females between 1 and 2 years of age (Figure 4a and 4c). Not surprisingly, this effect was weaker as the juveniles aged (Figures 4b and 4d). Combined survival estimates for suckling and non-suckling males and females were similar to those during the period of the decline to ages 1 and 2, but were greatly improved during the recent period for juveniles to ages 3 and 4 (Figure 5). Cumulative survival to age 4, when many females become reproductively mature [6], was 35.7±8.2% (SE).

Most juveniles were weaned by one year of age, but 16.9±2.2% of males and 22.6±1.8% of females were estimated to continue suckling between ages 1 and 2. Between ages 2 and 3, these proportions declined to 11.2±2.7% of males and 8.2±2.3% of females. Only one individual female and no males were observed to nurse beyond age 3. That particular female nursed through age 4 and gave birth for the first time to her own pup at age 5.

## Discussion

### Survival Comparisons Past and Present, East and West

Determining factors that affect juvenile survival is a fundamental problem for population ecologists. By comparing and contrasting the behaviors of conspecifics with differing population trends, we may gain some insight into mechanisms of variation in juvenile survival. Such mechanisms are ultimately the result of environmental influence but are often tempered through the quality and extent of maternal care [1], [3]. Western and eastern Steller sea lions provide an interesting study in contrast of the possible effects of differing maternal strategies as discussed below.

Survival from 3 weeks of age to 1 year was very high (nearly 80%) among WPDS sea lions in this study. This estimate is the same as it was during the period of the decline in this region [19] and generally better than in the EDPS where survival to 1 year was <60% at the largest and oldest rookeries and between 62% and 76% at 2 smaller, newer rookeries [23]. Even between ages 1 and 2, the combined estimates in this study for both suckling and non-suckling males and females were similar to estimates during the population decline in this region at about 57.5%, being diminished by the poor survival probability of weaned males in this age group. The improvement in survival over estimates during the decline in the WDPS seems to begin after age 2 with a jump to 89% in this study compared to 58% from earlier estimates [19]. Survival from age 2 and older was more similar between this study and current estimates from the EDPS [23] where populations have been increasing [24], [25]. Overall, cumulative survival to age 4 in this study was double (35.7%) the estimate during the decline in the WPDS (17.9%; [19], providing evidence that more females are recruiting into the breeding population in recent years. Food availability has been implicated as a primary contributor of survival to recruitment age among some pinnipeds [53], [54]. For Steller sea lions, a variety of factors including food availability and killer whale predation may affect recruitment [12], [55] and some of these are discussed in more detail below.

### The Effect of Mass

Among pinnipeds, survival has been correlated with pup mass and several maternal factors including parturition date, pupping location, and maternal age, experience and mass [28], [53], [56]–[60]. Notwithstanding differences between sexes, it is common among mammals for smaller individuals to have reduced chances of survival, especially during periods of greater resource competition or reduced food availability [28], [61]–[63]. In this study, birth mass was positively correlated with survival to 2 and 3 years of age among females and males that were weaned by age 1, but was unimportant for juveniles that continued to suckle past age 1. The same positive effect of mass at 2 – 4 weeks of age on survival through at least the first few years of life was found for EDPS Steller sea lions with the correlation diminishing for older animals [23]. However, pups that are smaller or grow more slowly may be able to compensate for their disadvantage by continuing to suckle later in life [7], [64]. Indeed, this study shows that mass was not an important contributor to survival for juveniles that continued to suckle beyond their first year of life.

### The Effect of Extended Maternal Care

Post-partum maternal care likely plays a greater role than birth mass in the future survival of offspring and this is believed to be true for phocids but more so for otariids with extended lactation periods [31], [65], [66]. Furthermore, large otariids such as the Steller sea lion give birth to relatively small young compared to smaller pinnipeds [67] making post-partum maternal care especially important in this species. Females that have difficulty transferring sufficient energy to their offspring risk mortality of the offspring or their own reduced fitness [29], [31], [68], [69]. Yet, pinnipeds that are able to adjust their lactation length are better adapted to changing environmental conditions [7] and this sort of adjustment can help offspring to reach a critical mass needed for weaning. Threshold mass and growth rates are believed to be the primary factors influencing the timing of weaning among large mammals [30], [64], [67]. It was not possible to measure weaning mass among Steller sea lions in this study, and birth mass was not found to contribute to the timing of weaning. Nevertheless, some interesting differences become apparent when comparing maternal investment and survival studies on a broader scale.

Mothers of pups in the Gulf of Alaska (WDPS) have longer perinatal periods and shorter foraging trips than mothers in the EDPS [32], [70], suggesting better maternal care early in life for WDPS pups. Furthermore, young pups have been found to be larger [71] and grow faster in both mass and size within the WDPS compared to the EDPS [72]. Although specific correlations have not been tested, it is reasonable to suggest that better maternal care early in life translates into better survival for WDPS sea lions through their first year compared to EDPS sea lions as explained herein. This study shows that continued maternal care has a positive influence on survival beyond 1 year of age. After their first year, offspring of EDPS mothers may be more likely to continue suckling, with as much as 70% observed doing so [8]. In contrast, only about 20% of WDPS juveniles suckle past age 1 with a corresponding large decrease in survival for individuals that were weaned. EDPS animals have better overall survival between ages 1 and 3 [23], which might be attributed to proportionally more juveniles continuing to suckle at older ages.

As pups are born heavier and grow faster in the WDPS, we can generalize that adult female sea lions in this region invest more in their offspring early in life and are able to wean them at an earlier age, whereas EDPS females provide less care early on but continue care for a longer period. This latter strategy is typical among otariids during times, and at locations, of low food availability [7], which may be the case for EDPS Steller sea lions. The population in the east is at the highest level seen in the past century [24] and likely subject to more intraspecific competition for resources compared to WDPS sea lions that are far below historical numbers. There is also some evidence that average age at weaning was increasing in the west between 1960 and 1983 in conjunction with a theorized reduction in food availability [73]. I suggest here that weaning age in the west has returned to base levels that are indicative of good food availability and that turnaround may have begun in the late 1980s – early 1990s as represented by good first-year survival during that time period [19]. However, some interannual variation in age at weaning may still persist [74], although it was not observed in this study. These comparisons between WDPS and EDPS Steller sea lions suggest that plasticity in the duration of maternal care is an important density dependent mechanism for populations at low and high levels of abundance respectively.

### Sex differences in survival

Differences in survival between juvenile male and female sea lions also provide an interesting study in contrasts. Juvenile males had lower survival probabilities than females to age 4 in this study. This was also the case for Steller sea lions in the expanding EDPS [23], but not during the WDPS decline [19]. Among pinnipeds, lower juvenile survival of males compared to females has also been observed in subantarctic fur seals (*Arctocephalus tropicalis*; [62]) and grey seals (*Halichoerus grypus*; [75]), but lacking in New Zealand sea lions (*Phocartos hookeri*; [76]), California sea lions (*Zalophus californianus*; [77]), and southern elephant seals (*Mirounga leonina*; [78]).

Hastings et al. [23] make several plausible arguments as to why male survival can be lower than female survival in Steller sea lions, including greater growth and maintenance requirements among males [79], a theory expounded by Clutton-Brock et al. [80]. The cost of physiological maintenance requirements in juvenile male Steller sea lions can be exacerbated by increased energy expenditure in more frequent, prolonged, and intense bouts of play behavior compared to females [81]. However, it should then follow that we might expect a further reduction in survival for males compared to females during times when high-quality food is less abundant [80] as it may have been during the period of the WDPS decline [15], [82], [83]. Yet, survival was differentially lower for females during the period of the decline compared to present day, whereas male survival to age 2 was actually better during the period of the decline than it has been in recent years ([19] vis-à-vis this study). Similar trends were found in subantarctic fur seals with females having a greater reduction in survival from years of good or average environmental productivity to years of poor productivity compared to males [62]. In these cases, the larger mass of males may provide some buffering against reduced resource availability as exemplified by the greater annual fluctuations in mass that males cope with compared to females [79]. Alternatively, males may be more persistent in suckling during times of reduced food availability [84]. This might explain their relatively better survival during the period of the decline and why proportionally more males than females suckle at older ages in the EDPS [8] compared to the WDPS (this study).

Explanations for sex differences in juvenile Steller sea lion survival during periods of good prey availability may include risks associated with greater travel distances by males [23], [85], and ‘incautious’ behavior of males leading to entanglements [86]. Energy expenditure associated with greater travel distances outside the normal home-range of subantarctic fur seals was implicated as a contributor to the higher mortality found in juvenile males [62]. Incautious behavior and broader travel ranges could also make males more susceptible to predation. Juvenile mortality in Steller sea lions has been strongly linked to predation in the eastern Gulf of Alaska with a greater proportion of males taken compared to females in a recent study, although the difference was not significant within the small sample [22]. Continued maternal care may temper imprudent behavior of juvenile males by providing increased vigilance or protection against predators. A similar effect of maternal vigilance on juvenile survival was also found in a predatory land mammal, the cheetah (*Acinonyx jubatus*; [87]).

Juvenile survival is an important element in the dynamics of populations and minor changes could have large impacts on pinniped populations [88]. High juvenile survival, coupled with recent high natality [21], may be important contributors to the recovery of the Steller sea lion population in the Gulf of Alaska following the catastrophic collapse in abundance throughout the Gulf of Alaska, Aleutian Islands, and Bering Sea. Survival likelihoods, and perhaps the primary causes of mortality, can differ between the sexes depending on interdecadal changes in food availability or predation pressure. The idea that Steller sea lions in much of the WDPS are doing better from a nutritional perspective than those in the EDPS in recent years is not new [12], [89]. However, this study offers new insight into how maternal care might affect the survival of different age classes of young sea lions and how adjustments can be made to ensure long-term success of the population. The results presented here should encourage further work into how variations in maternal care may provide some resilience to drastic population changes among long-lived mammals.
